# Friendship Network Characteristics Are Associated with Physical Activity and Sedentary Behavior in Early Adolescence

**DOI:** 10.1371/journal.pone.0145344

**Published:** 2015-12-28

**Authors:** Jennifer Marks, Kayla de la Haye, Lisa M Barnett, Steven Allender

**Affiliations:** 1 WHO Collaborating Centre for Obesity Prevention, Deakin University, Geelong, Australia; 2 School of Health and Social Development, Deakin University, Burwood, Australia; 3 Department of Preventive Medicine, University of Southern California, Los Angeles, California, United States of America; Cinvestav-Merida, MEXICO

## Abstract

**Introduction:**

There is limited understanding of the association between peer social networks and physical activity (PA), sedentary and screen-related behaviors. This study reports on associations between personal network characteristics and these important health behaviors for early adolescents.

**Methods:**

Participants were 310 students, aged 11–13 years, from fifteen randomly selected Victorian primary schools (43% response rate). PA and sedentary behaviors were collected via accelerometer and self-report questionnaire, and anthropometric measures via trained researchers. Participants nominated up to fifteen friends, and described the frequency of interaction and perceived activity intensity of these friends. Personal network predictors were examined using regression modelling for PA and sedentary/screen behavior.

**Results:**

Perceived activity levels of friends, and friendships with very frequent interaction were associated with outside-of-school PA and/or sedentary/screen time. Differences according to sex were also observed in the association between network characteristics and PA and sedentary time. A higher number of friends and greater proportion of same sex friends were associated with boys engaging in more moderate-to-vigorous PA outside of school hours. PA intensity during school-day breaks was positively associated with having a greater proportion of friends who played sports for girls, and a greater proportion of male friends for boys.

**Conclusion:**

Friendship network characteristics are associated with PA and sedentary/screen time in late childhood/early adolescence, and these associations differ by sex. The positive influence of very active peers may be a promising avenue to strengthen traditional interventions for the promotion of PA and reduction in screen time.

## Introduction

Few children meet recommended physical activity (PA) guidelines for optimal health benefits [1], and PA declines over childhood [2–5]. Sedentary behavior, which is often inversely related to PA but nonetheless qualitatively distinct, is also high and increasingly prevalent among children, particularly computer/TV screen time [6]. Both inadequate PA and excessive sedentary leisure time increase the risk of overweight, obesity and associated health risks in childhood [7, 8]. Further, these trends developed in childhood and adolescence track into adulthood [9, 10].

Emerging evidence exploring friendship influence on PA and sedentary behavior has identified a relationship between friends or broader peer groups and PA in late childhood/early adolescence [11, 12]. Peers are an integral, adaptive, and important influence on development and behavior throughout childhood [13]. Social influence theories suggest health-related behavior is influenced by a person's social context through various mechanisms such as peer modelling, imitation, and social learning [14]. Normative behavior (or perceptions thereof) is a further mechanism of social influence, whereby youth are motivated to adopt the typical behaviors of their peers to gain or retain group acceptance [15]. Social influence is also known to vary based on the depth of a relationship, with close friends often having more influence on an individual’s behavior than the broader peer group [16, 17]. Throughout childhood, a large proportion of these close friendships develop and flourish within the school environment. Social influence processes among friends (particularly close school friends) give rise to behavioral similarities among peers, although it is important to note that similarity may also be due to homophily; the tendency for youth to select and befriend others who are already similar to themselves in some way [18, 19]. The complex social mechanisms driving behavior in childhood and adolescence are important to understand [20]. This is particularly true of social contexts where childrens’ PA and sedentary behaviors take shape, to help inform PA interventions for altering or utilizing valuable social contingencies [21]. It is especially valuable to understand the role of peers in shaping these behaviors both at a time when peers become increasingly important referents for behavior from late childhood [22], and when PA is in decline.

Social network data captures a holistic perspective of social systems, such as how characteristics of the child and their social network partners collectively explain activity/sedentary behavior. Through examination of social network structure, composition, and social position, social network analyses explore relationships between an individual and particular network characteristics [23]. Evidence to-date demonstrates that childrens PA behavior is similar to that of their friends [24–26], particularly close/best friends [27–30], with peer influence on PA behavior generally stronger for boys than girls [12]. Research of friendship influence on sedentary behavior in late childhood/early adolescence is limited [11, 12], with findings of no association (age 12–18 years) [24], or differing by sex and/or network characteristic [28, 30]. Girls have been shown to have similar screen behavior within their friendship networks, while among males the evidence is mixed [28, 30]. Studies of peer influence on PA/screen behavior typically have not examined the strength of friendships beyond the distinction of either best/close friends or the broader peer group. For instance, close friends who see one another very frequently might conceivably influence each other’s behavior more than friends who interact occasionally.

Research on friendship associations with behavior is emerging, yet the complex characteristics of these relationships, and how they may differentially be associated with youth PA, is not well understood [11, 12, 20, 31]. Current evidence demonstrating relationships between friendships and PA has been conducted in varying social contexts and in different stages of childhood and adolescence [24, 26–30, 32–34]. More evidence on friends in relation to sedentary behavior is also needed. Further exploration of types of associations between networks and these activity behaviors is needed to advance our knowledge of mechanisms that impact youth PA, and to inform evidence-based health promotion intervention. The current study extends existing research by considering how these relationships hold when we look at more complex definitions of friendship, and more specific type of behavior.

The aim of this study was to explore cross-sectional associations between PA, sedentary behavior and friendship in late childhood/early adolescence. Being a cross sectional study, proposed mechanisms are based on theoretical perspectives and evidence to-date, but cannot be empirically tested. We explore PA and sedentary behavior associations with the size of friendship groups, frequency of interaction (inside and outside of school), and perceptions of others. Specifically, this was achieved by identifying nuanced personal friendship network characteristics (including number, frequency of interaction, and perceived activity level of friends), and through exploration of how these factors were associated with an individuals’ level of PA and sedentary behavior from late childhood. The secondary aim was to explore whether these associations differ according to sex. The investigation was based on: social norms theory where behavior is adopted of the broader group for peer acceptance; social facilitation theory where the presence of others affects individual behavior [35]; and social modelling theory where individuals imitate valued peers [13, 14].

## Methods

### Design and sample

Participants were 310 students (age 11–13 years) from 15 schools in Victoria, Australia. The sampling frame comprised all Victorian state government primary schools [36] stratified by a five-level indexed socio-economic scale. Schools were randomly selected from the bottom two socio-economic strata and invited to participate. Fifteen of twenty-seven schools approached (56%) provided written consent. The study was targeted towards year 6 students but one school region wished to involve more students, hence all students within year 6 at all schools and in year 5 at six schools, were invited to participate. Years 5 and 6 within these Australian schools represent the last years of primary schooling before the transition to secondary school commencing in year 7. Written parental consent was received for 43% (313/736) of invited students. The study was conducted in the 2013 final school term (Oct-Dec), three consenting students not available. Participation comprised completing a self-report behavioral and social network questionnaire, having anthropometric measurements taken, and wearing an accelerometer over a one week period. Methods other than the social network questionnaire have been described in detail elsewhere [37], and are outlined below.

This study was reviewed and approved by the Deakin University Human Research Ethics Committee (2013–093) and permission to approach schools was received from the Department of Education and Early Childhood Development (2013_001992) state school authority. Written informed parental consent was required for each study participant. Informed verbal consent from each participant was also obtained at the time of data collection. Students were assured they were free to withdraw or choose not to participate at any stage without any consequence. Strict confidentiality and documentation was maintained throughout the process of data collection and analysis.

### Self-report behavior

Self-report PA 5-point Likert scale and usual screen-time based questions [38] were collapsed into binary or categorical variables for analysis as follows. Recess and lunch PA intensity: 0) Low (sitting/walking); 1) Moderate-to- vigorous PA (MVPA) (playing/running). Physical education (PE) MVPA frequency: 0) Never/sometimes; 1) Quite often/always. Walking/cycling to/from school weekly frequency: 1) 0 times; 2) 1–5 times; and 3) 6–10 times. Self-report weekend MVPA: 0) <60 min/day; 1) ≥60 min/day. After-school MVPA: 0) <30 min/day; 1) ≥30 min/day, as a proxy for meeting/not meeting daily recommendations, derived using the after-school period from 3:30-6pm of 2.5 hours [39] as 50% of five hours of potential active time across the school day (0.25hr active transport to school, 1.25hrs recess and lunch periods, 1hr PE, 2.5hrs after-school). Average daily screen-time (weekdays and weekends) were categorized as either meeting or not meeting ≤ 2hrs/day guideline. Screen-time was capped at a maximum of 8 hours per day [40].

### Personal friendship networks

Participants listed the full names, school-year level, and sex of up to fifteen friends they “hang around with the most”, and whether the friend attends their school. For each friend, they also reported on the times/settings in which they “hang around” (recess, lunch, after school, and/or weekends), used to compute a summary frequency of interaction score (from 0–4). “Very frequent interaction” (score of 3–4) was defined to denote friendships that involve interactions both inside and outside of school. Participants also indicated whether they usually played sport with each friend ‘more than once a week’, and their perception of how active their friend was [1) *not very active (doesn’t do sport or exercise very often at all);* 2) *sometimes active (once or twice a week);* 3) *very active (most days)*].

Summary network variables were computed to represent the total number of nominated friends (network out-degree), and percentages of total nominated friends with the following characteristics: same sex as participant; same school-year level; not attending the same school; play sport with; perceived PA level; and interaction time/setting (recess, lunch, afterschool, weekends). Multiplex variables, summarizing multiple dimensions of a participant’s social network characteristics, were derived for the proportion of friends: (1) with very frequent interaction, (2) with very frequent interaction who were also “very active”, and (3) at recess, lunch, after-school or weekends who were very active. Distributions of these network characteristics, reported as medians, were often heavily skewed towards 0% or 100%. Due to the limited data variability in some network characteristics in this sample, many of which are typical of this age group (e.g. tendencies to have predominantly same sex friends), some variables were recoded as binary. In instances where ≥ 75% participants reported 0% of a characteristic (‘% not same school’, ‘% very frequent interaction’, ‘% very active friends of frequent interaction’, ‘% very active after-school friends’, ‘% very active weekend friends’, ‘% sometimes active’ and ‘% not very active’), the network variable was recoded as ‘0’ meaning *0% of the network or* ‘1’ meaning *a percentage greater than a null value*. Where ≥ 75% of participants reported networks with 100% of a particular characteristic (‘% same sex’ and ‘% in same year level’), the network variable was recoded as ‘1’ meaning *100% of the network or* ‘0’ meaning *a percentage less than 100% of the network*. All other network characteristics that were represented as percentages showed greater variability, namely ‘% play sport with’ and ‘% very active’, and so were recoded into an equal interval 10-point scale, and rounded to whole numbers.

### Anthropometrics

Due to the relationship between BMI and the behaviors of interest, and evidence of an effect between BMI and friendship selection and influence [41], weight status was calculated and controlled for to accurately identify network associations with PA and sedentary behavior. Height and weight were measured using calibrated Charder HM200P height stadiometers and A&D UC-321 weight scales according to standardized protocols [42]. Average measures were used for analysis. Body Mass Index (BMI; weight in kg/(height in m)^2^) and standardized scores were calculated using the WHO reference 2007 Stata module. Weight status was defined using WHO age-specific BMI cut-offs, and categorized as either “under/normal weight” (BMI ≤ 1 standard deviation (SD)) or “overweight/obese” (BMI > 1 SD) [43].

### Accelerometry

Objective PA data was collected via ActiGraph GT1M accelerometer (ActiGraph LCC, Pensacola, US) [44], recorded at 15 second epochs and analyzed using ActiLife 6 software. Minimum wear time was defined as 480 minutes over any three 8 hour days, non-wear time as >60 minutes of consecutive zero counts with a 2-minute tolerance [45, 46]. Intensity cut points were defined as: sedentary (<1.5 metabolic equivalents (METs); ≤ 100 counts/min); light PA (> = 1.5 to <4 METs); and MVPA (> = 4 METs) [47]. MVPA was further categorized as “<60 min/day” and “≥60 min/day”.

### Statistical analyses

Demographic, behavior and personal network descriptive analyses were conducted by gender from the compiled dataset (S1 Dataset). To account for clustering (nesting) of individuals, multilevel regression models were fit using a two-level approach at the school and individual level. To test specific personal network characteristic associations (out-degree, % very frequent interaction, % very active and % same sex) with PA/sedentary behavior, each variable was initially included within regression models. The network variable ‘% play sport with’ was also tested in each model, as an important characteristic identified within other network studies [12]. Where two or more variables were highly correlated (*r* > 0.6), (e.g. multiplex variables and their originally derived variable), only one variable was included within the same model. Non-statistically significant exploratory variables were dropped from models if their effect size was small, or had little effect on the model. These included the variables: % not same school, % in same year level, and % very active friends of frequent interaction. Little variation at the school level from initial models were identified, however final multilevel models were fit to account for any potential clustering effect. Final models were also adjusted for age and categorical weight status, with separate analyses by sex. Objective PA models were also adjusted for accelerometer wear time. Linear, logistic and Poisson regression were used to model continuous, dichotomous and count outcome variables respectively. Final regression models are shown separately for males and females for each dependent variable. All exploratory variables were retained within each model unless their inclusion resulted in non-statistical significance. Due to the low response rate within some schools, we focus on personal network characteristics and not complete network characteristics (e.g. participant position within the school friendship network), as the latter approach requires complete or near-complete response rates. All analyses were conducted in 2015 using Stata 12.0 software (StataCorp LP, College Station, US).

## Results

### Descriptive characteristics

Descriptive statistics are provided in Table 1. Mean age of participants was 12.1 years: 129 male (42%); 181 (58%) female. Thirty-eight percent of participants (44% male; 34% female) were overweight or obese. Accelerometer derived measures and most self-report behaviors show males to be more physically active than females. Proportionately more males engaged in higher levels of screen time on weekdays compared to females. No significant differences were found by sex for self-report MVPA after school, PE active frequency, walking and cycling to school, or weekend recreational screen time.

**Table 1:** Participant characteristics: demographic and behavior.

	All	Male	Female	
	(*n* = 310)	(*n* = 129)	(*n* = 181)	*p*>
Demographic				
Age, years				
Mean (SD)	12.1 (0.5)	12.1 (0.6)	12.0 (0.5)	0.12
School level				
Year 5, n (%)	65 (21)	31 (24)	34 (19)	0.26
Year 6, n (%)	245 (79)	98 (76)	147 (81)	
Weight status^1^				
Under/normal weight, n (%)	190 (62)	72 (56)	118 (66)	0.09
Overweight/obese, n (%)	117 (38)	56 (44)	61 (34)	
*Missing*, *n*	*3*	*1*	*2*	
Behavior: Physical activity				
MVPA by recommendation^1^				
< 60 min/day, n (%)	184 (73)	58 (57)	126 (83)	**<0.01**
≥ 60 min/day, n (%)	69 (27)	43 (43)	26 (17)	
*Missing*, *n*	*57*	*28*	*29*	
Average daily (min) MVPA^1^				
Mean (SD)	51 (19)	58 (20)	46 (17)	**<0.01**
Average daily (min) light PA^1^				
Mean (SD)	221 (42)	229 (44)	216 (40)	**0.02**
MVPA after school^2^				
< 30 min/day, n (%)	94 (31)	38 (30)	56 (32)	0.73
≥ 30 min/day, n (%)	209 (69)	89 (70)	120 (68)	
*Missing*, *n*	*7*	*2*	*5*	
MVPA on weekends^2^				
< 60 min/day, n (%)	138 (45)	45 (35)	93 (52)	**<0.01**
≥ 60 min/day, n (%)	171 (55)	84 (65)	87 (48)	
*Missing*, *n*	*1*	*0*	*1*	
PE very active frequency^2^				
Never or sometimes, n (%)	72 (23)	23 (18)	49 (27)	0.06
Quite often or always, n (%)	237 (77)	105 (82)	132 (73)	
*Missing*, *n*	*1*	*1*	*0*	
Activity level at recess^2^				
Low (sitting, walking), n (%)	144 (47)	40 (32)	104 (58)	**<0.01**
Moderate to vigorous, n (%)	162 (53)	87 (69)	75 (42)	
*Missing*, *n*	*4*	*2*	*2*	
Activity level at lunch^2^				
Low (sitting, walking), n (%)	124 (41)	32 (25)	92 (52)	**<0.01**
Moderate to vigorous, n (%)	182 (59)	96 (75)	86 (48)	
*Missing*, *n*	*4*	*1*	*3*	
Walk to/from school^2^				
0 times/week, n (%)	155 (50)	65 (50)	90 (50)	0.87
1–5 times/week, n (%)	98 (32)	42 (33)	56 (31)	
6–10 times/week, n (%)	57 (18)	22 (17)	35 (19)	
Cycle to/from school^2^				
0 times/week, n (%)	230 (74)	92 (71)	138 (76)	0.09
1–5 times/week, n (%)	58 (19)	23 (18)	35 (19)	
6–10 times/week, n (%)	22 (7)	14 (11)	8 (4)	
Behavior: Sedentary / screen time				
Average daily (min) sedentary^1^				
Mean (SD)	470 (76)	452 (68)	481 (79)	**<0.01**
Usually watch TV/videos/DVDs^2^				
Yes, n (%)	271 (88)	111 (86)	160 (89)	0.37
Usually play non-active computer games^2^				
Yes, n (%)	129 (42)	69 (53)	60 (33)	**<0.01**
Usually use computer for leisure^2^				
Yes, n (%)	161 (52)	57 (44)	104 (57)	**0.02**
Recreational screen time (weekday)^2^				
≤ 2 hours/day, n (%)	196 (64)	73 (57)	123 (69)	**0.04**
> 2 hours/day, n (%)	111 (36)	55 (43)	56 (31)	
*Missing*, *n*	*3*	*1*	*2*	
Recreational screen time (weekend)^2^				
≤ 2 hours/day, n (%)	179 (59)	67 (52)	112 (63)	0.06
> 2 hours/day, n (%)	127 (42)	61 (48)	66 (37)	
*Missing*, *n*	*4*	*1*	*3*	

MVPA, moderate to vigorous physical activity; PA, physical activity; PE, physical education; SD, standard deviation

^1^. Objectively measured;

^2^. Self-report measure

p, test value for sex differences using Pearson χ2 test for equality of percentages or t-test of means; boldface indicates statistical significance (p<0.05)

Personal friendship network descriptive statistics are given in Table 2. Mean network out-degree was 6.1 (males: 5.5; females: 6.5; *P* < 0.05). Almost all nominated friends were of the same sex as the participant, with very few not from the same school. Recess and lunch were times of high interaction with nominated friends. Participants had few (median = 13%) ‘friends with very frequent interaction’ (friends both inside and outside of school). Participants rated at least half (median: males 75%; females 50%) of their social networks as ‘very active’, while very few (median = 0%) were perceived as ‘not very active’. Compared to females, males had a significantly higher average percentage of friends they played sport with, spent recess and lunch times with, and who were perceived as very active. Females reported a greater proportion of friends who were perceived as being ‘sometimes active’ compared to males (median: females 33%; males 13%)

**Table 2:** Participant characteristics: personal friendship networks.

	**All (*N* = 310)**			**Male (*n* = 129)**			**Female (*n* = 181)**			
	**Mean**	**SD**	**Range**	**Mean**	**SD**	**Range**	**Mean**	**SD**	**Range**	***p***
Total friends nominated (outdegree)	6.1	3.2	0–15	5.5	2.8	1–15	6.5	3.4	0–15	**<0.01**
Personal network characteristics	**Median**	**Q1**	**Q3**	**Median**	**Q1**	**Q3**	**Median**	**Q1**	**Q3**	***p***
% of same sex friends	100	93	100	100	100	100	100	90	100	0.38
% not at the same school	0	0	0	0	0	0	0	0	13	**<0.01**
% in same year level	71	50	100	75	50	100	70	50	100	0.66
% usually play sport with	71	29	100	83	33	100	63	20	100	**<0.01**
Time/location of interaction with friends										
% at recess	100	73	100	100	88	100	89	67	100	**<0.01**
% at lunch	90	67	100	100	77	100	83	60	100	**<0.01**
% after school	10	0	33	0	0	33	14	0	33	0.14
% on weekends	14	0	40	11	0	40	17	0	40	0.31
Perceived friend PA behavior										
% very active	60	33	100	75	40	100	50	25	85	**<0.01**
% sometimes active	29	0	56	13	0	50	33	0	65	**<0.01**
% not very active	0	0	0	0	0	0	0	0	7	0.10
Multiplex relational characteristics										
% friends with very frequent interaction ^1^	13	0	33	13	0	50	12	0	33	0.28
% friends with very frequent interaction who are very active^1^	0	0	21	0	0	25	0	0	17	**0.02**
% friends at recess who are very active	50	21	80	67	33	100	36	15	67	**<0.01**
% friends at lunch-time who are very active	45	20	75	60	25	100	33	11	60	**<0.01**
% after school friends who are very active	0	0	20	0	0	25	0	0	18	0.79
% weekend friends who are very active	0	0	22	0	0	25	0	0	20	0.58

PA, physical activity; Q, quartile (Q1-Q3 interquartile range); SD, standard deviation

^p^, test value for sex differences using t-test of means or Wilcoxon rank sum test of medians; boldface indicates statistical significance (p<0.05)

^1^. Frequency of interaction at least 3 of 4 from recess, lunch, after school or weekends

### Physical activity

Regression models are shown separately for each dependent PA variable and by gender in Table 3. Personal network characteristics that were statistically significant predictors of an individual’s PA generally differed for males and females.

**Table 3:** Regression models for personal network characteristic predictors of PA for males and females.

**Males**	**MVPA min/day**			**After school MVPA SR**			**Weekend MVPA SR**		
	**(accelerometer)**			**(≥ 30 min/day)**			**(≥ 60 min/day)**		
	Coef	95% CI	^p^	OR	95% CI	*p*	OR	95% CI	*p*
Total friends (*outdegree*)	-0.56	-1.97, 0.85	0.44	1.33	1.09, 1.62	**<0.01**	1.38	1.15, 1.66	**<0.01**
% of same sex friends^1^	2.01	-6.86, 10.87	0.66	1.49	0.45, 4.93	0.52	4.43	1.53, 12.8	**<0.01**
% usually play sport with^2^	0.29	-0.85, 1.43	0.62	0.96	0.82, 1.13	0.60	0.97	0.85, 1.11	0.70
% very active^2^			*NS*	1.08	0.93, 1.25	0.31	0.98	0.85, 1.12	0.74
% very frequent interaction ^3^ ^,^ ^4^			*CV*	3.01	1.13, 8.01	**0.03**	2.92	1.24, 6.86	**0.01**
% very active after school friends^3^	9.37	1.71, 17.03	**0.02**			*CV*			*CV*
**Females**	**MVPA min/day**			**After school MVPA SR**			**Weekend MVPA SR**		
	**(accelerometer)**			**(≥ 30 min/day)**			**(≥ 60 min/day)**		
	No significant predictors			OR	95% CI	*p*	OR	95% CI	*p*
Total friends (*outdegree*)				1.09	0.97, 1.23	0.14	1.06	0.96, 1.18	0.26
% of same sex friends^1^						*NS*	0.78	0.36, 1.69	0.53
% usually play sport with^2^				1.15	1.03, 1.28	**0.01**	1.09	1.00, 1.20	0.06
% very active^2^				1.08	0.97, 1.21	0.18	1.14	1.03, 1.26	**0.01**
% very frequent interaction ^3^ ^,^ ^4^						*NS*	2.36	1.24, 4.49	**<0.01**
**Males**	**Recess MVPA**			**Lunch time MVPA**			**Cycle to/from school**		
	**(SR rating)**			**(SR rating)**			**(no. of times)**		
	OR	95% CI	*p*	OR	95% CI	*p*	OR	95% CI	*p*
Total friends (*outdegree*)	1.04	0.86, 1.25	0.69	1.16	0.96, 1.40	0.12	1.08	0.91, 1.28	0.37
% of same sex friends^1^	8.22	2.29, 29.54	**<0.01**	4.71	1.45, 15.30	**0.01**	2.17	0.66, 7.10	0.20
% usually play sport with^2^	1.02	0.88, 1.18	0.84	1.12	0.97, 1.29	0.11			*NS*
% very active^2^	1.09	0.94, 1.27	0.23	1.07	0.92, 1.24	0.37			*NS*
% very frequent interaction ^3^ ^,^ ^4^	2.14	0.79, 5.82	0.14	2.65	0.95, 7.37	0.06			*NS*
% very active after school friends^3^			*CV*			*CV*	3.66	1.54, 8.71	**<0.01**
**Females**	**Recess MVPA**			**Lunch time MVPA**			**Cycle to/from school**		
	**(SR rating)**			**(SR rating)**			**(no. of times)**		
	OR	95% CI	*p*	OR	95% CI	*p*	No significant predictors		
Total friends (*outdegree*)	0.96	0.86, 1.07	0.43	0.96	0.85, 1.08	0.47			
% of same sex friends^1^	0.74	0.34, 1.61	0.44	0.52	0.23, 1.18	0.12			
% usually play sport with^2^	1.11	1.00, 1.22	**0.04**	1.14	1.03, 1.26	**0.01**			
% very active^2^	1.06	0.95, 1.17	0.28	1.06	0.95, 1.18	0.29			
% very frequent interaction ^3^ ^,^ ^4^			*NS*	1.03	0.52, 2.04	0.93			

OR, odds ratio; 95% CI, 95% confidence interval; MVPA, moderate to vigorous physical activity; PA, physical activity; SR, self-report

CV, correlated with other variable within model; NS, model not significant if variable included

All models controlled for individual level (age and weight status) and school level covariates. Objective measured PA models controlled for accelerometer wear time.

p, test value, boldface indicates statistical significance (p<0.05)

^1^. Binary variable recoded as either 100% or less than 100%;

^2^. 10-point scale variable;

^3^. Binary variable coded as either 0% or greater than 0%;

^4^. Frequency of interaction at least 3 of 4 from recess, lunch, after school or weekends

#### Friendship number and frequency of interaction

For males, as out-degree friendship nominations increased, the odds of achieving at least 30 min/day MVPA (self-report) after-school (OR = 1.33; 95% CI = 1.09, 1.62) or 60 min/day on weekends (OR = 1.38; 95% CI = 1.15, 1.66) increased. Having friendships that entailed frequent interaction was also positively associated with time spent engaging in MVPA (self-report) on weekends for males (OR = 2.92; 95% CI = 1.24, 6.86) and females (OR = 2.36; 95% CI = 1.24, 4.49), and after-school for males only (OR 3.01; 95% CI = 1.13, 8.01).

#### More active friends

For females, engaging in at least 60 min/day MVPA (self-report) on weekends was positively associated with the proportion of their friends perceived as being ‘very active’ (OR = 1.14; 95% CI = 1.03, 1.26). Females were also more likely to engage in MVPA (self-report) at recess (OR = 1.11; 95% CI = 1.0, 1.22) and lunch (OR = 1.14; 95% CI = 1.03, 1.26), and for at least 30 min/day after-school (OR = 1.15; 95% CI = 1.03, 1.28) if they had a higher proportion of friends they usually ‘played sport with’. For males, having a greater proportion of after-school friends who were also very active was associated with an increase in objectively measured MVPA (9 min/day; 95% CI = 1.71, 17.03) and a higher frequency of cycling to/from school (OR = 3.66; 95% CI = 1.54, 8.71).

#### Friends of the same sex

For males only, having a higher proportion of male friends increased the likelihood of engaging in MVPA (self-report) at recess (OR = 8.22; 95% CI = 2.29, 29.54) and lunch (OR = 4.71; 95% CI = 1.45, 15.30), and for at least 60 min/day on weekends (OR = 4.43; 95% CI = 1.53, 12.80).

#### No association

Physical activity outcomes that *were not* significantly predicted by personal network characteristics, after adjusting for age and weight status, included: (1) PE activity intensity, and (2) light PA (objective) for males and females; and (3) objective MVPA and (4) walking/cycling to/from school for females.

### Sedentary behavior

For females, spending more than two hours/day in recreational screen time on weekends was inversely associated with the proportion of having ‘very active’ friends (OR = 0.87; 95% CI = 0.78, 0.96), as presented in Table 4. For males, a higher proportion of friends perceived as ‘sometimes active’ was predictive of more sedentary time (24 min/day).

**Table 4:** Regression models for personal network characteristic predictors of sedentary (males) and weekend-screen time (females).

**Males**	**Sedentary min/day**		
	**(accelerometer)**		
	Coef	95% CI	*p*
Total friends (*outdegree*)	0.91	-2.82, 4.64	0.63
% of same sex friends^1^	-0.66	-23.21, 21.89	0.95
% sometimes active^2^	24.35	4.46, 44.23	**0.02**
% very frequent interaction ^2^ ^,^ ^3^	-9.05	-28.43, 10.33	0.36
**Females**	**Weekend screen time**		
	**(> 2 hrs/day)**		
	OR	95% CI	*p*
Total friends (*outdegree*)	1.13	1.02, 1.25	**0.02**
% very active^4^	0.87	0.78, 0.96	**<0.01**
% very frequent interaction ^2^ ^,^ ^3^	0.69	0.35, 1.36	0.29

All models controlled for individual level (age and weight status) and school level covariates. Objective measured sedentary time models controlled for accelerometer wear time.

OR, odds ratio; 95% CI, 95% confidence interval

p, test value, boldface indicates statistical significance (p<0.05)

^1^. Binary variable recoded as either 100% or less than 100%;

^2^. Binary variable coded as either 0% or greater than 0%;

^3^. Frequency of interaction at least 3 of 4 from recess, lunch, after school or weekends;

^4^. 10-point scale variable

No network characteristics were found as predictors of: (1) weekday recreational screen time (all); (2) weekend recreational screen time (males); or (3) sedentary time (females).

## Discussion

Type and intensity of friendship, and the time and context of interaction with friends were associated with different behaviors, many of these differing by gender. Time spent engaging in PA was positively associated with having higher: numbers of friends; proportion of friends with frequent interaction; and proportion of friends perceived as being very active. An inverse relationship between participant screen time and the proportion of their friends that were very active friends was also found. A unique aspect of this study was the exploration of relational nuances of where and when friends interact, offering a temporal and contextual perspective of which relationships matter to PA and sedentary behavior from late childhood.

Previous research suggests that close friends who are also active provide youth with opportunities for PA engagement and behavior modelling [27], where close friends’ PA behaviors are adopted and become increasingly similar over time [29]. Contrary to expected [27], we did not find increased frequency of interaction among friends, likely indicative of friendship intensity/‘closeness’, to be more prominently associated with overall PA than other friendship network predictors. Having very active close (of frequent interaction) friends was not an important PA predictor over and above having very active friends. In their study of late childhood, Jago et al. [27] found best friends MVPA to be predictive of participant MVPA, whilst de la Haye et al. [28] found the broader close friend network was associated with PA engagement. Similarly, the current study found MVPA associations among the broader identified friendship group, and close friends as further differentiated within the network (high frequency of interaction), but unlike Jago et al.[27] and de la Haye et al.[28], these associations were not dependent upon MVPA of the best/close friend. It is possible that MVPA of best/close friends were similar within the current study, however other network characteristics such as out-degree and gender were also shown to be predictive of individual MVPA. Together these studies of different friendship characteristics demonstrate the potential for friends to influence and reinforce normative PA behavior. Results also suggest that whilst close friends are important, social influence from friends through modelling or adopting normative behaviors is no more prominent among close friends than the broader friendship group. This implies that interventions, rather than focusing on close friend behavior modelling, should consider targeting whole friendship groups, as it is the larger group normative behaviors that are important. Efforts to promote PA should also consider how to provide PA opportunities for those who have few close friends, or who are socially marginalized from their peers.

Few associations with objectively measured MVPA were evident within the current study. Despite this, using nuanced personal network characteristics we were able to tease out associations with PA at various times of the day/week using self-reported measures. It could be that participants have similar biases for perceptions of their own PA and that of their friends, inflating the association between these variables. However results are consistent with evidence of positive associations between friends and objectively derived MVPA outside of school hours in childhood [27, 33], identifying potential areas to target for increasing overall MVPA throughout the week day.

Although having more friends had no impact on average daily MVPA, the likelihood that boys spent ≥ 60 minutes/day in MVPA on weekends, and ≥ 30 minutes after school, increased with more friends. Previous research has identified the size of children/adolescents’ friendship groups [28] and the proportion of friends they play sports with [24] to be positively associated with PA. Together these findings suggest that having more friends increases opportunity for PA engagement (likewise, social isolation/less friends reduces opportunity) [48] particularly outside of school-hours, such as team-based structured sporting activity.

There is evidence that gender of friends is an important factor that may impact peer influence on adolescent PA, with friend similarity on PA level evident among same-sex friends [26, 28, 30]. Within the current study, having female friends was not associated with females engaging in MVPA, but for males, having more male friends was significant for MVPA. In contrast, a large US based study with adolescents (average age 14 years) found MVPA in females to be positively associated with their male and female friends, and MVPA in males to be positively associated with their female (but not male) friends [26]. Although different in sample size and geographic region, both studies had higher PA reported by males, similar levels of overweight status in boys and girls, and a similar number of friends nominated by participants. The main difference was the friendship characteristic tested. Sirard et al. [26] found MVPA time was positively predicted by their friends MVPA, dependent upon the gender of the friend, as important dyad-level characteristics for influencing PA. The current study, which does not focus on PA homophily and gender between friends, provides additional insight into the role of gender and MVPA within relationships that have varying levels of intensity and interaction contexts, by examining how characteristics of social networks as a whole, including gender composition and other factors, are relevant to adolescent PA. Together, these results demonstrate the importance of considering both gender and the context of friendship groups when targeting increased PA in early adolescence.

In the current study, playing sport with friends was a significant factor for girls (but not boys) engaging in more MVPA time after school, and increasing PA intensity at school recess and lunch times. In contrast, a recent study found no association between PA and girls (slightly younger, aged 10–11) playing sport with friends [27]. It could be that early adolescence is an important time in the development of friendship influence on individual behavior, and suggests that promoting structured activity may be an effective strategy for encouraging PA in girls at this age.

Consistent with evidence that PA and sedentary behavior frequently co-exist [49], we found that boys’ sedentary time was positively associated with a perception of their friends being ‘sometimes active’. Similar to previous research [24, 28], we also found no evidence of association between boys’ friendship networks and screen time, whereas higher out-degree and a lower proportion of very active friends were predictive of higher screen time on weekends for girls. Sex differences in personal network associations appear to reflect the nature of sedentary/screen behavior. Whilst the majority of participants watched TV/videos/DVDs, boys were more likely than girls to play non-active computer games and spend more than two hours screen-time per day. In contrast, girls were more likely to engage in internet/social media usage. These results also suggest that having more active friends not only encourages PA participation, but provides social interaction outside of the school environment that may displace other recreational pursuits like screen-based recreational time.

### Strengths and limitations

A major study strength was the use of both objective and self-report PA measures that enabled identification of different friendship associations within and outside of school contexts. Self-report was also a limitation, introducing potential risk of respondent bias. Participants’ perceptions of their friends’ behavior may also have bias. Yet inherent with perceptions of social norms theory [50], the study focus was whether perceptions, not the accuracy of assessment, were associated with behavior. The low participant response rate did not permit analysis of complete network characteristics such as in-degree, density or reciprocity. Instead, conducting ego (personal network) analyses provided the advantage of broadening the scope of friendships to those outside of school, an area currently understudied, which also enabled frequency of interaction with friends to be explored, giving insight into nuances of adolescent social behavior patterns. An additional limitation was the inability to determine underlying mechanisms (e.g. social influence/selection) leading to the observed network-behavior associations, due to the cross-sectional design. Further longitudinal research is needed to advance this knowledge.

### Conclusion

This research suggests that PA and sedentary behaviors are associated with complex characteristics of children’s peer social environments. During late childhood/early adolescence peers are an important influence on behavior and these social mechanisms should be important aspects of intervention design to increase PA and reduce sedentary behaviors. These patterns are gendered particularly regarding timing and types of activity. Interventions might seek to provide opportunities and environments (e.g. parks, recreational facilities) for engaging in PA/sport with friendship groups, or to normalize being very active for both girls and boys.

## Supporting Information

S1 Dataset Physical activity, sedentary behavior and network characteristics dataset. (CSV)
